# Medical status of patients presenting for treatment at an Australian dental institute: a cross-sectional study

**DOI:** 10.1186/s12903-020-01285-2

**Published:** 2020-10-21

**Authors:** Agnieszka M. Frydrych, Richard Parsons, Omar Kujan

**Affiliations:** 1 UWA Dental School, The University of Western Australia, 17 Monash Avenue, Nedlands, WA 6009 Australia; 2 School of Occupational Therapy, Social Work and Speech Pathology, Curtin University, Bentley, WA 6102 Australia

**Keywords:** Systemic diseases, Dentistry, Dental institute, Western Australia, Cross-sectional

## Abstract

**Background:**

Individuals seeking dental treatment often present with medical conditions which may affect dental treatment provision. The purpose of this study was to determine the prevalence of medical conditions and medication use among individuals attending dental clinics at a Western Australian tertiary institution.

**Methods:**

This observational study examined the general demographics, medical and social histories of 873 subjects presenting for a general dental examination at a Western Australian Tertiary Institution between March 2018 and February 2019. Individual recruited were those presenting to the clinics either as new patients to the Centre or returning patients after an extended period of absence.

**Results:**

Presence of a medical condition was reported by 86% of the participants, with males more commonly affected (*p* = 0.0448). Medication use was noted by 80% of the participants. Cardiovascular disease (37.9%), allergy (32.3%), mental health disorders (29.4%), musculoskeletal (23.0%), endocrine (22.4%) and respiratory disease (17.8%) were the most common conditions identified. Women were significantly more likely to report allergy (*p* = 0.0002) or a mental health condition (*p* = 0.0368).

**Conclusion:**

Medical comorbidities are common among individuals presenting for student dental treatment in Western Australia, highlighting the importance of knowledge and teaching of internal medicine and its application to the dental setting.

## Background

Individuals seeking dental treatment often present with underlying medical conditions, which may or may not be managed with a variety of medications [1–16]. These comorbidities impact on the delivery of dental treatment and the importance of understanding the health status of the dental patient is well recognised [17–19]. The safety and effectiveness of dental treatment provision rests on the degree to which an individual’s medical status is considered in dental treatment planning. This consideration in turn is highly dependent on the clinician’s knowledge of internal medicine and its application to the dental setting. Shortcomings of the dental curricular with respect to the preparation of students to deal with patients with underlying medical conditions have been demonstrated in a number of studies [20, 21].

Although grounding in general medicine is essential for the dental students in the modern era; understanding the most commonly encountered medical conditions in the dental setting, has the potential to shift the focus of internal medicine teaching. This may motivate students to learn internal medicine through interest and appreciation of relevance of subjects discussed. Focusing teaching around student experience may also help the students to better contextualize their theoretical knowledge [22].

A number of studies have been undertaken with the aim of ascertaining the types of medical conditions prevalent among dental patients in general [1, 8, 10] and specialist practice [9, 10, 12, 14, 15] as well as in dental school patient populations [2, 3, 5–7, 9–14, 16]. The prevalence of medical problems among dental patients has been shown to vary greatly, ranging from 1 to 83%, depending on the population studied [1–3, 6–10, 12–16]. Elderly patients and those attending public dental facilities have been shown to demonstrate increased prevalence of systemic disease compared to their younger counterparts or those seeking treatment in the private sector [10, 12, 13]. While a broad range of comorbidities has been reported, cardiovascular disease [5–10, 12–14, 16], respiratory conditions [2, 3, 5, 7, 8, 10–12, 14, 16], endocrine disorders [1–3, 6, 7, 10–14, 16], bleeding pathosis [1, 5, 11], arthritis [2, 6–8, 11–13, 16], and allergies [1, 2, 6, 7, 9–11, 13, 16] constitute the most commonly reported medical problems encountered in the dental settings. Despite fewer studies reporting medication use, the most commonly prescribed medications parallel the most frequently encountered medical problems, with medications used in the management of cardiovascular disease the most commonly reported [6, 7, 13, 15].

At present there is a general paucity of Australian data [8, 10] and a lack of data concerning the medical status of patients seen by the dental students in Western Australia. In view of this, the aims of this study were to 1. determine the prevalence of medical conditions and medication use among dental patients attending the general dental clinics for a routine dental examination at the Oral Health Centre of Western Australia (OHCWA), University of Western Australia and 2. present associations between patient demographics and social habits and subject’s medical history and medication use. The prevalence estimates are documented and compared between males and females because differences in some of these are known to exist between genders [2, 5, 7, 13, 14]. It is anticipated that results of this study will shed light on the types of comorbidities of dental patients encountered by the dental students during their clinical studies, thus stimulating discussion around internal medicine content delivery, with the view to better prepare the graduates to deal with the medically compromised patients.

## Methods

This was an observation, cross-sectional study based on examination of existing patient records approved by the Human Research Ethics Committee of the University of Western Australia (RA/4/20/4435) and has been reported in accordance with the Strengthening the Reporting of Observational Studies in Epidemiology (STROBE) statement [23].

Study participants were recruited from a pool of patients presenting to the general dental clinics at the Oral Health Centre of Western Australia (OHCWA), University of Western Australia between March 2018 and February 2019. Individual recruited were those presenting to the clinics either as new patients to the Centre or returning patients after a period of absence of at least 12 months. From the administrative and assessment perspective, patients representing to the Centre for treatment after a period of absence are treated the same as new patients. The institution represents the only centralised public tertiary referral centre in Western Australia, where eligible patients are treated on behalf of the State Government. Attendance at the OHCWA is governed by strict eligibility criteria based on an individual’s socioeconomic status, which are checked each time an individual attends the Centre. For an individual to continue to receive treatment at the Centre, they must continue to meet the eligibility criteria. All new patients and those representing after a period of absence seeking to obtain treatment at the Centre undergo a dental examination and a comprehensive assessment of their medical and social history, which is recorded in the Centre’s Titanium Oral Health Management (TOHM) system. We focused on new patients presenting to the Centre as this allowed us to collect the subjects’ most comprehensive and up to date social and medical history data and minimized the problem of incomplete or inaccurate records. Throughout the study period, we regularly audited the clinicians to ensure that the patient history was accurately and fully recorded. All new patients presenting to the Centre (and those returning after a period of absence) during the study period were enrolled in the study with the choice to opt-out from participation. Participation was voluntary, and participants were at liberty to withdraw from the study at any time, with no disadvantage to their ongoing treatment.

The study subjects were individuals aged 25 years or older attending the OHCWA general dental clinics for a routine dental examination. All individuals with unknown medical history or opting out from participation in the study were excluded. The recorded variables for each subject were derived from the data recorded in TOHM and included: age, gender, residential post code, IRSAD (socioeconomic status score, based on residential postcode), medical history (including drug history) as well as smoking and alcohol use.

### Statistical analysis

Following record de-identification, all data were manually entered into a Microsoft Excel spreadsheet (Redmond, WA, USA) and verified independently to prevent data transfer error. The data were then exported into SAS version 9.4 (SAS Institute Inc, Cary, NC, USA 2008) for analysis. Simple descriptive analyses (frequencies and percentages) were used to summarise the demographic and medical profile of the study participants. Chi-square statistics were used to compare these profile variables between male and female participants (univariate analyses). In comparing the two genders, odds ratios for females compared to males were calculated for each variable, along with its 95% confidence interval. The mean IRSAD was compared between genders for medical history and medication use, using the Student’s t-test. Statistical significance was set at *p* < 0.05 in all tests.

## Results

One thousand and four subjects were approached for inclusion in the study. A total of 131 were excluded from the study, either because they chose not to participate or their medical history was incomplete, so that a total of 873 subjects were included in the final analysis. The general characteristics of all participants, and divided according to gender, are presented in Table 1. Male subjects were more likely to use tobacco (*p* < 0.0001) and consume alcohol (*p* = 0.0002). Eighty six percent of study participants reported having a medical condition, with males more likely to be affected (*p* = 0.0448).

**Table 1:** General characteristics of the participants

Variable	Total N = 873	Male N = 394 (%)	Female N = 479 (%)	*p* value (Chi-square test)
Age group (years)				0.4519
25–34	105	40 (10.2)	65 (13.6)	
35–44	131	54 (13.7)	77 (16.1)	
45–54	137	61 (15.5)	76 (15.9)	
55–64	147	66 (16.8)	81 (16.9)	
65–74	228	112 (28.4)	116 (24.2)	
75–84	103	52 (13.2)	51 (10.7)	
85+	22	9 (2.3)	13 (2.7)	
Tobacco use				< 0.0001
Never	481	168 (42.6)	313 (65.5)	
Current	201	109 (27.7)	92 (19.3)	
Ex-user	190	117 (29.7)	73 (15.3)	
Tobacco (current/ex-user)				0.5061*
Self-rolled	19	10 (4.4)	9 (5.5)	
Cigarette	370	214 (94.7)	156 (94.6)	
Chew/vape/other	2	2 (0.9)	0 (0.0)	
Alcohol use				0.0002
Never	457	177 (44.9)	280 (58.6)	
Current	393	203 (51.5)	190 (39.8)	
Ex-user	22	14 (3.6)	8 (1.7)	
Alcohol by type				0.0001
Spirits	25	14 (9.3)	11 (9.1)	
Wine	133	56 (37.1)	77 (63.6)	
Beer	59	45 (29.8)	14 (11.6)	
Cider	6	3 (2.0)	3 (2.5)	
Mixed	49	33 (21.9)	16 (13.2)	

The *p* value compares each variable between male and female respondents

*Fisher’s Exact test

Table 2 illustrates the prevalence of disease and medication usage among the study participants. Cardiovascular disease (37.9%), allergy (32.3%), mental health disorders (29.4%) musculoskeletal (23.0%) endocrine (22.4%) and respiratory disease (17.8%) constituted the most common conditions reported. Women were significantly more likely to report allergy (*p* = 0.0002) or a mental health condition (*p* = 0.0368). No gender differences were observed amongst the remaining commonly reported pathosis.

**Table 2:** The prevalence of disease and medication usage among the cases

Variable	Total N = 873 (%)	Males N = 394 (%)	Females N = 479 (%)	Odds ratio (Female relative to Male) (95% CI)	*p* value (Chi-square test)
*Disease characteristics*					
Presence of disease	753 (86.3)	350 (88.8)	403 (84.1)	0.67 (0.45–0.99)	0.0448
Allergy	282 (32.3)	102 (25.9)	180 (37.6)	1.72 (1.29–2.31)	0.0002
Auditory system disorder	11 (1.3)	5 (1.3)	6 (1.3)	0.99 (0.30–3.26)	1.0*
Blood disorder	23 (2.6)	6 (1.5)	17 (3.6)	2.38 (0.93–6.09)	0.0629
Cancer	100 (11.5)	48 (12.2)	52 (10.9)	0.88 (0.58–1.33)	0.5402
Cardiovascular disease	331 (37.9)	153 (38.8)	178 (37.2)	0.93 (0.71–1.23)	0.6124
Gastrointestinal system disorder	118 (13.5)	51 (12.9)	67 (14.0)	1.09 (0.74–1.62)	0.6537
Endocrine system disorder	196 (22.4)	81 (20.6)	115 (24.0)	1.22 (0.88–1.68)	0.2241
Diabetes	136 (15.6)	63 (16.0)	73 (15.2)	0.94 (0.65–1.36)	0.7611
Dyslipidaemia	38 (4.4)	15 (3.8)	23 (4.8)	1.27 (0.66–2.48)	0.4736
Other endocrine	49(5.6)	10 (2.5)	39 (8.1)	3.40 (1.68–6.91)	0.0003
Immune system disorder	38 (4.4)	19 (4.8)	19 (4.0)	0.82 (0.43–1.56)	0.5377
Zoonotic diseases	15 (1.7)	13 (3.3)	2 (0.4)	0.12 (0.03–0.55)	0.0011
Liver disease	15 (1.7)	7 (1.8)	8 (1.7)	0.94 (0.34–2.61)	0.9041
Mental health disorder	257 (29.4)	102 (25.9)	155 (32.4)	1.37 (1.02–1.84)	0.0368
Musculoskeletal system disorder	201 (23.0)	92 (23.4)	109 (22.8)	0.97 (0.70–1.33)	0.8355
Nervous system	108 (12.4)	51 (12.9)	57 (11.9)	0.91 (0.61–1.36)	0.6409
Renal system	29 (3.3)	13 (3.3)	16 (3.3)	1.01 (0.48–2.13)	0.9733
Reproductive system	35 (4.0)	9 (2.3)	26 (5.4)	2.46 (1.14–5.30)	0.0185
Respiratory system disorder	155 (17.8)	68 (17.3)	87 (18.2)	1.06 (0.75–1.51)	0.7280
Asthma	5 (10.9)	39 (9.9)	56 (11.7)	1.21 (0.78–1.86)	0.3974
COPD	32 (3.4)	18 (4.6)	14 (2.9)	0.63 (0.31–1.28)	0.1978
Other	41 (4.7)	17 (4.3)	24 (5.0)	1.17 (0.62–2.21)	0.6287
Skin conditions	26 (3.0)	14 (3.6)	12 (2.5)	0.70 (0.32–1.53)	0.3646
Viral infections	48 (5.5)	26 (6.6)	22 (4.6)	0.68 (0.38–1.22)	0.1957
Visual system disorder	35 (4.0)	16 (4.1)	19 (4.0)	0.98 (0.50–1.92)	0.9436
Other disease	5 (0.6)	1 (0.3)	4 (0.8)	3.31 (0.37–29.73)	0.3854*
*Medication usage*					
Taking Medication	699 (80.0%)	317 (80.5)	382 (79.9)	0.97 (0.69–1.35)	0.8421
Ace inhibitor	86 (9.9%)	51 (12.9)	35 (7.31)	0.53 (0.34–0.83)	0.0054
Alpha Blockers	5 (0.6)	4 (1.0)	1 (0.2)	0.20 (0.02–1.83)	0.1811*
Analgesics	150 (17.2)	59 (15.0)	91 (19.0)	1.33 (0.93–1.91)	0.1168
Opioid	69 (7.9)	36 (9.1)	33 (6.9)	0.74 (0.45–1.20)	0.2206
Non-opioid	106 (12.1)	34 (8.6)	72 (15.0)	1.87 (1.22–2.88)	0.0040
Antiarrhythmic	7 (0.8)	2 (0.5)	5 (1.0)	2.07 (0.40–10.71)	0.4668*
Anticoagulants	150 (17.2)	84 (21.3)	66 (13.8)	0.59 (0.41–0.84)	0.0033
Anticonvulsants and antiepileptics	73 (8.4)	27 (6.9)	46 (9.6)	1.44 (0.88–2.37)	0.1440
Antidepressants	197 (22.6)	65 (16.5)	132 (27.6)	1.93 (1.38–2.69)	0.0001
SNRI	39 (4.5)	15 (3.8)	24 (5.0)	1.33 (0.69–2.58)	0.3918
SSRI	79 (9.0)	23 (5.8)	56 (11.7)	2.14 (1.29–3.54)	0.0027
Tricyclic	25 (2.9)	7 (1.8)	18 (3.8)	2.16 (0.89–5.22)	0.0807
Combination	22 (2.5)	10 (2.5)	12 (2.5)	0.99 (0.42–2.31)	0.9754
Other	32 (3.7)	10 (2.5)	22 (4.6)	1.85 (0.86–3.95)	0.1079
Antihistamine	19 (2.2)	9 (2.3)	10 (2.1)	0.91 (0.37–2.27)	0.8466
Antihypertensive NOS	38 (4.3)	24 (6.1)	14 (2.9)	0.46 (0.24–0.91)	0.0224
Anti-inflammatories	74 (8.5)	25 (6.4)	49 (10.2)	1.68 (1.02–2.78)	0.0403
Corticosteroid	26 (3.0)	10 (2.5)	16 (3.3)	1.33 (0.60–2.96)	0.4878
NSAID	51 (5.8)	16 (4.1)	35 (7.3)	1.86 (1.01–3.42)	0.0419
Antiemetics	11 (1.3)	2 (0.5)	9 (1.9)	3.76 (0.81–17.5)	0.1238*
Antimicrobial	35 (4.0)	15 (3.8)	20 (4.2)	1.10 (0.56–2.18)	0.7825
Antipsychotic	60 (6.9)	27 (6.9)	33 (6.9)	1.01 (0.59–1.70)	0.9830
Beta blockers	91 (10.4)	45 (11.4)	46 (9.6)	0.82 (0.53–1.27)	0.3817
Bisphosphonates	9 (1.0)	3 (0.8)	6 (1.3)	1.65 (0.41–6.65)	0.5242*
Calcium channel blockers	55 (6.3)	31 (7.9)	24 (5.0)	0.62 (0.36–1.07)	0.0838
Chemotherapeutics	7 (0.8)	4 (1.0)	3 (0.6)	0.61 (0.14–2.76)	0.7072*
Diuretics	34 (3.89)	18 (4.6)	16 (3.3)	0.72 (0.36–1.43)	0.3506
Anxiety and sleep disorders	66 (7.6)	30 (7.6)	36 (7.5)	0.99 (0.60–1.63)	0.9563
Drugs for Diabetes	105 (12.0)	55 (14.0)	50 (10.4)	0.72 (0.48–1.08)	0.1115
Drugs for dyslipidaemia	181 (20.7)	98 (24.9)	83 (17.3)	0.63 (0.46–0.88)	0.0062
Statin	156 (17.9)	85 (21.6)	71 (14.8)	0.63 (0.45–0.90)	0.0096
Fibrate	15 (1.7)	8 (2.0)	7 (1.5)	0.72 (0.26–1.99)	0.5197
Other	18 (2.1)	8 (2.0)	10 (2.1)	1.03 (0.40–2.63)	0.9528
Gastrointestinal NOS	9 (1.0)	5 (1.3)	4 (0.8)	0.66 (0.17–2.46)	0.5276
Genitourinary	23 (2.6)	18 (4.6)	5 (1.0)	0.22 (0.08–0.60)	0.0012
H2 antagonists	9 (1.0)	4 (1.0)	5 (1.0)	1.03 (0.27–3.86)	1.0*
Hormones	111 (12.7)	23 (5.8)	88 (18.4)	3.63 (2.25–5.87)	< 0.0001
Immunosupressants	36 (4.1)	17 (4.3)	19 (4.0)	0.92 (0.47–1.79)	0.7969
Inhaled	106 (12.1)	42 (10.7)	64 (13.4)	1.29 (0.85–1.96)	0.2240
Laxatives	10 (1.1)	6 (1.5)	4 (0.8)	0.54 (0.15–1.94)	0.3600*
Muscle relaxants	5 (0.6)	3 (0.8)	2 (0.4)	0.55 (0.09–3.29)	0.6626*
Nitrates and anti-anginal	18 (2.1)	9 (2.3)	9 (1.9)	0.82 (0.32–2.08)	0.6749
Other drugs affecting bone	21 (2.4)	4 (1.0)	17 (3.6)	3.59 (1.20–10.75)	0.0150

*Fisher’s Exact test

Medication use was reported by 80% of the study participants with the pattern of medication usage paralleling the medical conditions reported. Antidepressants were the medications most commonly used by the individuals presenting for treatment with 22.6% of all subjects reporting the use of such medications. Women were twice as likely to report taking antidepressant medication compared to their male counterparts (*p* = 0.0001; odds ratio [95% CI] 1.93 [1.38–2.69]). Analgesics (17.2%), anticoagulants (17.2%) and drugs for dyslipidaemia (20.7%) were also commonly used in the population studied.

The effect of age group, gender, smoking and alcohol use on reporting a positive medical history and medication use is shown in Table 3. Young males (age group 25–34) were significantly more likely to report a positive medical history compared to females in the same age group (*p* = 0.0069). Among the current tobacco users, men were significantly more likely to present with a medical condition compared to their female counterparts (*p* = 0.0340). Among the non-drinkers, more men than women described having a medical condition (*p* = 0.0383). Compared to males (Table 4), women from lower socioeconomic background were significantly less likely to use medication (*p* = 0.0298).

**Table 3:** The effect of age group, gender, smoking and alcohol use on positive medical history and medication use

Variable	Male N = 394 (%)	Female N = 479 (%)	*p* value (Chi-square test)
Medical history			
Age group (years)			
25–34	34/40 (85.0)	39/65 (60.0)	0.0069
35–44	41/54 (75.9)	60/77 (77.9)	0.7890
45–54	51/61 (83.6)	63/76 (82.9)	0.9118
55–64	60/66 (90.9)	70/81 (86.4)	0.3972
65–74	106/112 (94.6)	108/116 (93.1)	0.6284
75–84	50/52 (96.2)	51/51 (100.0)	0.4951*
85+	8/9 (88.9)	12/13 (92.3)	1.0*
Medication			
Age group (years)			
25–34	27/40 (67.5)	36/65 (55.4)	0.2185
35–44	35/54 (64.8)	52/77 (67.5)	0.7458
45–54	43/61 (70.5)	58/76 (76.3)	0.4415
55–64	53/66 (80.3)	68/81 (84.0)	0.5643
65–74	101/112 (90.2)	107/115 (93.0)	0.4358
75–84	50/52 (96.2)	50/51 (98.0)	1.0*
85+	8/9 (88.9)	11/13 (84.6)	1.0*
Medical history			
Tobacco use			
Never	147/168 (87.5)	259/313 (82.8)	0.1708
Current	99/109 (90.8)	74/92 (80.4)	0.0340
Ex-user	104/117 (88.9)	69/73 (94.5)	0.1859
Medication			
Tobacco use			
Never	130/168 (77.4)	244/313 (78.2)	0.8355
Current	87/109 (79.8)	75/92 (81.5)	0.7607
Ex-user	100/117 (85.5)	62/73 (84.9)	0.9189
Medical history			
Alcohol use			
Never	158/177 (89.3)	230/280 (82.1)	0.0383
Current	181/203 (89.2)	164/190 (86.3)	0.3891
Ex-user	11/14 (78.6)	8/8 (100.0)	0.2727*
Medication			
Alcohol use			
Never	145/177 (81.9)	222 (79.6)	0.5370
Current	161/203 (79.3)	151/190 (79.5)	0.9681
Ex-user	11/14 (78.6)	8/8 (100.0)	0.2727*

*Fisher’s Exact test

**Table 4:** The effect of IRSAD^a^ (mean, standard deviation, number) on positive medical history and medication use

Variable	Male (N = 394)	Female (N = 479)	*p* value (Chi-square test)
Medical history			
No	1035 [60.1], 44	1025 [64.7], 76	0.4117
Yes	1038 [64.3], 350	1031 [67.3], 403	0.1335
Medication			
No	1029 [63.7], 77	1034 [67.6], 96	0.6230
Yes	1040 [63.7], 317	1029 [66.8], 382	0.0298

^a^IRSAD—The Index of Relative Socio-Economic Advantage and Disadvantage (Australian Bureau of Statistics)

## Discussion

Individuals seeking dental treatment often present with medical comorbidities, which affect the safety and effectiveness of dental treatment provision. Clinician’s knowledge of internal medicine and its application to the dental setting is therefore paramount [6]. This begins with and is facilitated by appropriate dental curriculum content delivery [22]. The immense and ever-increasing medical knowledge poses challenges to the complete coverage of the field in the dental curriculum [24]. While endless amounts of medical facts are unlikely to significantly improve the general care of the dental patient, the challenge is to adequately prepare students to manage patients with medical problems within the teaching time available. Focus of internal medicine teaching of both dental students and graduates on the most commonly encountered medical conditions aids the education process [22]. Demonstration of relevance of internal medicine teaching and importance of relevant case-based instruction has been emphasized in the literature [25]. This approach is reliant on the understanding of patient comorbidities the dental students are likely to encounter.

To date, little is known about medical comorbidities and medication usage of Australian dental patients. In this study we presented a uniquely detailed analysis of the medical status of patients seen by the dental students in Western Australia. We reported a high prevalence of systemic diseases in our study population, which can be attributed to the age of the participants and their association with a public institution. This is in keeping with other studies [10, 12, 13], which have also shown that older patients and those attending public dental facilities exhibit increased prevalence of systemic disease compared to their younger counterparts or those seeking treatment in the private sector. Eligibility at the OHCWA is defined by lower socioeconomic status, which in turn is linked to poorer health [26].

Cardiovascular disease (37.9%), allergy (32.3%), mental health disorders (29.4%) musculoskeletal (23.0%) endocrine (22.4%) and respiratory disease (17.8%) constituted the most common conditions reported with the pattern of medication usage paralleling the diseases reported. This is in keeping with other studies [1–16] and also reflects the most common chronic diseases in Australia [27]. Each of those conditions and associated medication use can not only contribute to the presentation of numerous intraoral pathosis but also affect the safe delivery of dental treatment. While dental treatment may potentially exacerbate the underlying medical condition; underlying medical conditions may also increase the risk of dental treatment complications such as bleeding or infection. Gender differences were observed only with respect to allergy and mental health disorders, with women more commonly affected. This is consistent with Australian data, where women experience a higher prevalence of mental health disorders then men [28]. Women were also twice as likely to take antidepressant medication.

Medication use was reported by 80% of study participants. This is significant given the potential for dental treatment complications and drug interactions. Of note, 17% of study participants reported taking anticoagulants, with men significantly more likely to be affected (*p* = 0.0033; odds ratio [95% CI] 0.59 [0.41–0.84]). Although this figure is significantly higher than is reported in the literature [6, 7], it is not unexpected give the high prevalence of cardiovascular and nervous system disease in the population studied. Our study participants were also specifically questioned about bleeding during medical history taking and were therefore potentially more likely to recall taking such medication. Drug interactions are an important consideration in dentistry [29–31]. Interactions between adrenaline used in dental local anaesthetics and medications such as tricyclic antidepressants and betablockers are well recognized [29]. In our study 1 in 10 participants reported the use of such medication. Antimicrobials used in dentistry such as erythromycin, clarithromycin, azolic antifungal agents and metronidazole affect the cytochrome P450 system by interacting with various substrates including CYP3A4, CYP2C9 and CYP1A2 [30]. This leads to potentially serious interactions with numerous medications including antihistamines, anticoagulants, antiepileptics, antidepressants, antipsychotics, anticonvulsants, antiviral agents, drugs for dyslipidaemia, calcium channel blockers, immunosuppressants, opiates and benzodiazepines [30]. Many of these were commonly taken by our study population (Table 2). Analgesic agents including paracetamol, selective and non-selective non-steroidal anti-inflammatory drugs (NSAIDs) and narcotics are often prescribed in the dental setting. Opiates exhibit additive sedative and respiratory depressor effects with other central nervous system depressors [31]. They also react with selective serotonin reuptake inhibitors and serotoninergic medication [31]. Given that in our study 23% of study participants reported using antidepressant medications, these are important interactions for dentist to be aware of. Prescription of NSAIDs in hypertensive patients treated with angiotensin-converting enzyme (ACE) inhibitors, beta-blockers, diuretics and angiotensin receptor antagonists can increase bleeding after surgery [31]. One in 10 of our study participants reported using ACE inhibitors and 1 in 10 beta-blockers. The interaction between paracetamol and warfarin [31] is also important to remember in view of the prevalent use of anticoagulants by dental patients.

Aside from treatment complications and drug interactions, medication usage is additionally associated with numerous adverse effects on oral health [32]. These include (but are not limited to): xerostomia and salivary gland hypofunction, dysgeusia, angioedema, mucosal discolouration, gingival hyperplasia, ulceration, erythema multiforme and lichenoid drug reactions [32]. Majority of the medications taken by our study participants have been linked to some type of an adverse oral health affect [32]. Given the prevalence of medication usage among dental patients, the importance of adequate teaching of pharmacology to the dental students cannot be overstated.

Teaching internal medicine and its relevance to the dental setting requires in-depth knowledge of both disciplines and can be challenging. Interdisciplinary exposure is therefore emphasized [25]. Engagement of medical and dental clinicians in the teaching of the most common medical problems encountered in the dental setting can facilitate the most efficient use of time and resources and will broaden the students’ educational experience.

Our study was based on examination of patient records generated from information supplied by the participants and is therefore limited by the accuracy of data recorded. Consideration should also be given to a potential selection bias. Although we have minimized the study burden on our participants by employing an opt-out option from participation, it is possible that the healthier individuals were more likely to make the effort to opt-out from the study. Finally, our study is limited to the fact it was based on a population derived from a tertiary referral centre providing subsidized dental services to the members of the public who meet eligibility criteria governed by their socioeconomic status. Results of this study should therefore be considered in the setting of the lower socioeconomic status of the study participants compared to the general Western Australian population. While the population presented in our study is more likely to present with medical conditions and report medication use compared to the wider population that a general dentist is likely to encounter in a private practice setting; it does illustrate the type of medical problems the dental students and dentists are likely to encounter and must be able to manage. It highlights areas on which the internal medicine teaching should focus, to ensure that dental students are well placed to confidently manage patients upon graduation. Finally, this study brings to light the broad experience opportunities that the dental students encounter and the teaching opportunities that exist in a Western Australian tertiary referral centre. Identification of the commonly encountered medical conditions by the dental students has the potential to lay foundation for an integrated case-based program of internal medicine which enhances students’ interest and study motivation through demonstration of relevance.

## Conclusion

This study presented a uniquely detailed analysis of the medical status of individuals presenting for dental treatment to a Western Australian tertiary referral centre. Eighty six percent of the participants reported having a medical condition with 80% taking at least one medication. Cardiovascular disease, allergy, mental health disorders, musculoskeletal, endocrine and respiratory disease constituted the most common conditions reported, highlighting the importance of the clinicians’ knowledge of internal medicine and its application to the dental setting.

## Data Availability

The data that support the findings of this study are available on reasonable request from the corresponding author. The data are not publicly available due to privacy or ethical restrictions.

## Abbreviations

ACE: Angiotensin-converting enzyme
IRSAD: Index of Relative Socio-economic Advantage and Disadvantage
NSAID: Non-steroidal anti-inflammatory drugs
OHCWA: Oral Health Centre of Western Australia
STROBE: The Strengthening the Reporting of Observational Studies in Epidemiology
